# Cleaning Up the Chimney: Early Renal Stent Graft Thrombosis Following Endovascular Treatment of a Juxtarenal Aortic Aneurysm With the Chimney Technique

**DOI:** 10.7759/cureus.54669

**Published:** 2024-02-22

**Authors:** Ioakeim Giagtzidis, Marios Theologou, Ioakeim Papoutsis, Christos Karkos, Konstantinos Papazoglou

**Affiliations:** 1 5th Surgical Department, Hippokrateio General Hospital, Aristotle University of Thessaloniki, Thessaloniki, GRC; 2 Vascular Surgery, 5th Surgical Department, Hippokrateio General Hospital, Aristotle University of Thessaloniki, Thessaloniki, GRC

**Keywords:** endovascular fenestration, renal stent thrombosis, chimney technique, endovascular aneurysm repair, juxtarenal aortic aneurysm

## Abstract

The endovascular management of juxtarenal aortic aneurysms with the chimney technique (ch-EVAR) has gained popularity in recent years. It provides an alternative to open repair, allowing treatment of challenging anatomies with devices readily available in any vascular suite. The primary drawback persists as the occurrence of type-Ia endoleak from gutters and renal stent thrombosis.

We present two cases of early renal stent graft thrombosis following chimney endovascular aneurysm repair. The first patient was an 80-year-old man who underwent single ch-EVAR and came back on the fifth post-op day with renal stent graft thrombosis. He was re-operated for recanalization and additional stenting of his chimney graft. The patient recovered well with no complications.

The second case involved a 72-year-old man with a juxtarenal aneurysm, treated with ch-EVAR on both renal arteries. Unfortunately, on the 10th post-op day, he was referred to our department due to lumbar pain and acute renal failure due to chimney graft thrombosis bilaterally. The left renal chimney graft was recanalized by endovascular means. On the contrary, despite efforts of the endovascular and open approach, the right chimney graft and the right renal artery remained occluded.

While ch-EVAR is a viable and off-the-shelf solution for urgent and complex juxtarenal aortic aneurysms, it should be performed with awareness of the potential for graft thrombosis and persistent endoleaks. Despite these complications, the chimney technique can still be a viable treatment option. A better understanding of the indications and advancements in the devices used can lead to better long-term results.

## Introduction

The chimney endovascular technique (ch-EVAR) was initially presented as a last resort therapeutic approach in an emergency setting, such as acute and complex pararenal or juxtarenal aortic pathologies or in an advert event of renal artery coverage during an EVAR [1]. Since then, it has gained huge popularity because standard EVAR has replaced open aneurysm repair and customized fenestrated or branched grafts are frequently unavailable, especially in an emergency setting. Their main indication is for pararenal or thoracoabdominal aneurysms. They also have increased costs [2]. However, this technique attracts a lot of critique due to gutter leaks responsible for type Ia endoleak and cases of early chimney graft thrombosis that can result in kidney failure [3].

The purpose of this case report is to present two cases of juxtarenal abdominal aortic aneurysms (AAA) treated with the chimney technique that unfortunately developed early chimney graft thrombosis, which was treated by endovascular means.

## Case presentation

**Figure 1:**
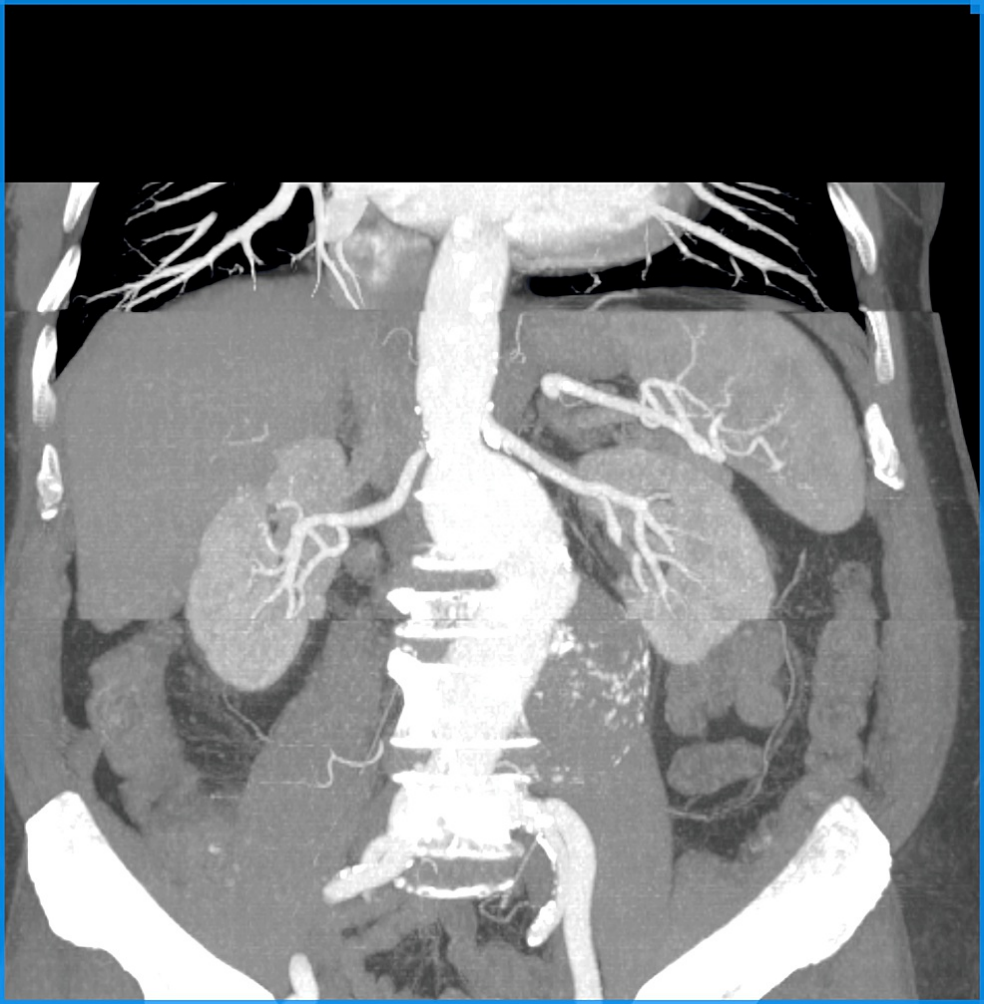
Case 1: Preoperative CTA. CTA, computed tomography angiography

In the first case, an 80-year-old male with incidental finding of an abdominal juxtarenal aortic aneurysm with a max sack diameter of 78 mm was referred to our department (Figure 1). His past medical history involved hypertension, coronary artery disease with an episode of myocardial infarction three years ago, and an episode of transient ischaemic attack a year ago.

A standard EVAR with a right renal artery chimney was decided due to a 3mm proximal aortic neck below the right renal artery orifice. The operation took place 24 hours after his admittance. All endovascular procedures were performed in a vascular hybrid theater, equipped with a mobile Siemens Arcadis C-Arm (Siemens Healthineers, Erlangen, Germany).

**Figure 2:**
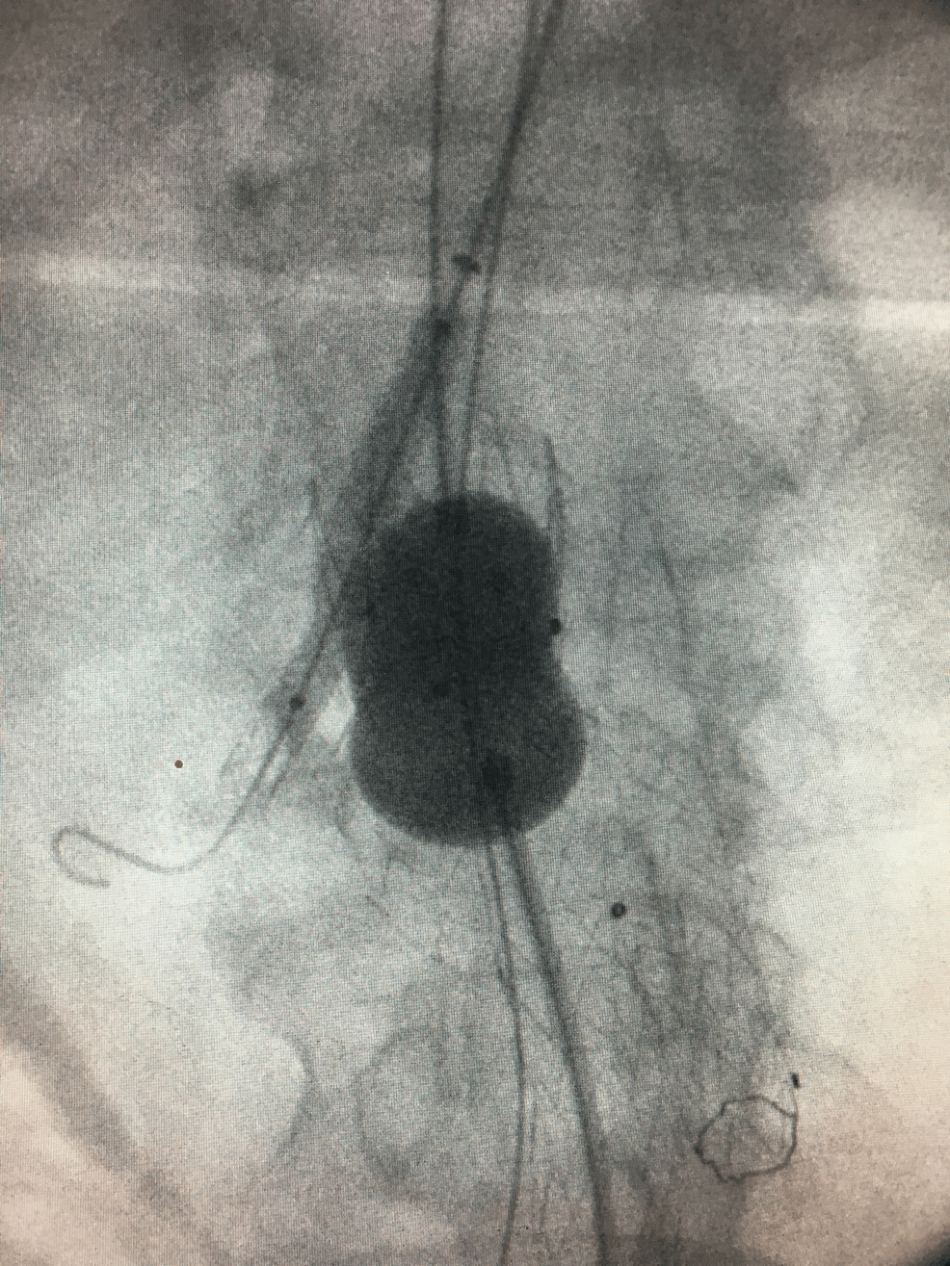
Case 1: Kissing balloon of the main graft and the renal stent graft.

The patient was placed in the supine position, with his left arm extended with external rotation. Under local anesthesia, both common femoral and left axillary arteries were dissected. Through direct arterial puncture, a 6 Fr 65 cm sheath (arrow) was inserted via the left axillary artery, and a 7 Fr 45 cm sheath (Arrow) was introduced through the left femoral artery, facilitated by the use of a stiff 0.035” hydrophilic angled wire. A 5 Fr H-H angiographic catheter was employed for cannulating the right renal artery via the arm. Subsequently, a balloon-expandable Bentley 7 mm x 40 mm stent graft (Innomed GmbH, Germany) was delivered and deployed following a wire exchange using a super stiff *J* tipped with a short 5 cm standard segment. An Endurant II 36-20-166 mm stent graft (Medtronic Vascular Inc., Santa Rosa) main body was inserted from the right femoral artery and deployed 1 cm proximal of the orifice of the right renal artery with an additional iliac extension (16-20-124 mm). A *Reliant* molding balloon was used, while the Begraft’s delivery balloon was simultaneously inflated (kissing balloon technique) (Figure 2).

**Figure 3:**
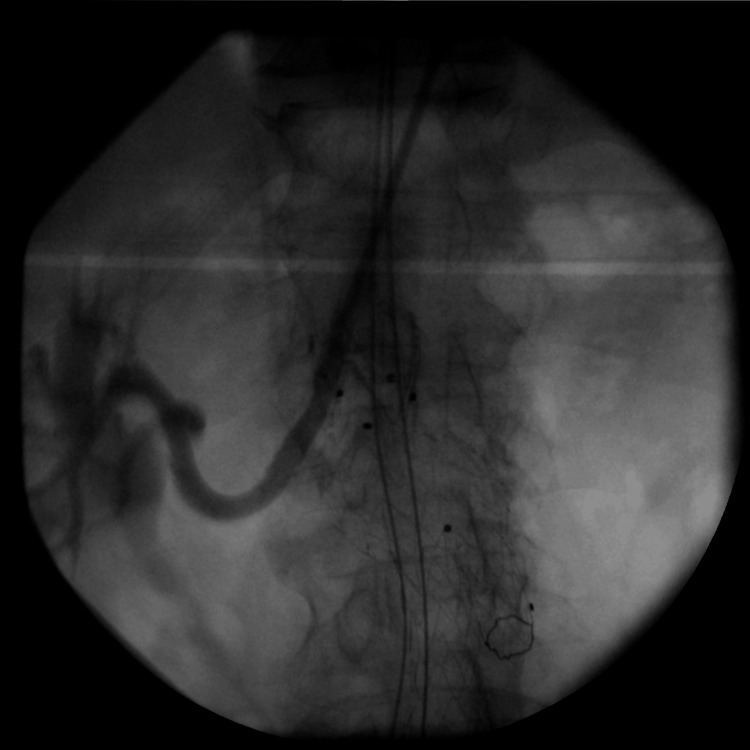
Case 1: Final angiography of the right renal chimney.

The contralateral limb was cannulated with the help of a *cobra* catheter, and a 16-20-156 mm graft was successfully deployed in the left common iliac artery. Final angiography showed good renal and graft patency with no endoleak (Figure 3). The fluoroscopy time for the duration of the procedure was 28 minutes and 40 seconds, with a total contrast amount of 250 mL administered. The patient had an uneventful recovery and was discharged on the third postop day with a prescription of clopidogrel 75 mg once daily and normal renal function.

**Figure 4:**
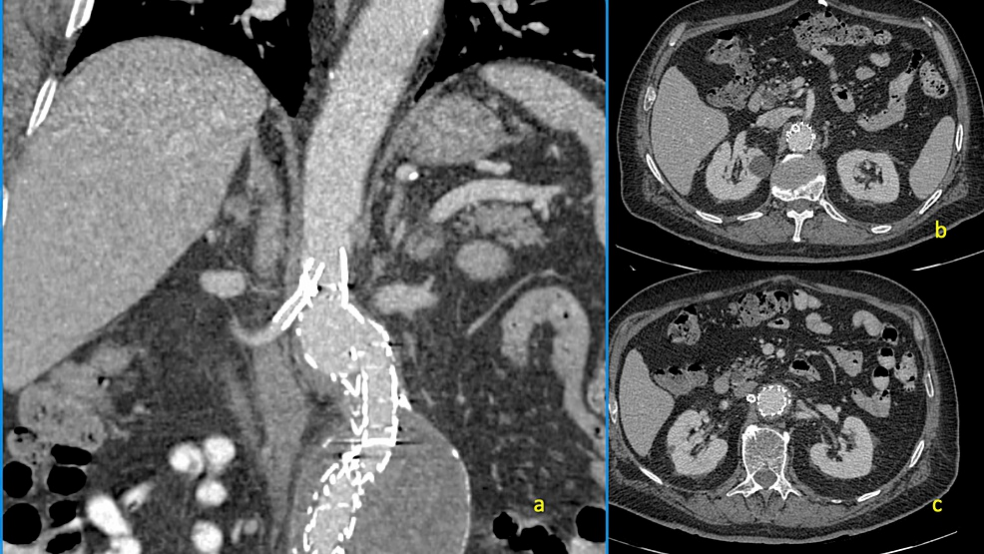
Case 1: Postop CTA. (a)-(c) Right renal stent graft chimney thrombosis. CTA, computed tomography angiography

During the fifth postop day, while at home, the patient experienced acute right lumbar pain. A new CTA conducted at a district hospital revealed right renal thrombosis without clear signs of kinking, compression, or obstruction of the stent graft and demonstrated slow post-occlusion flow in the distal renal artery (Figure 4). The renal function remained unaffected with normal values of plasma creatinine levels.

**Figure 5:**
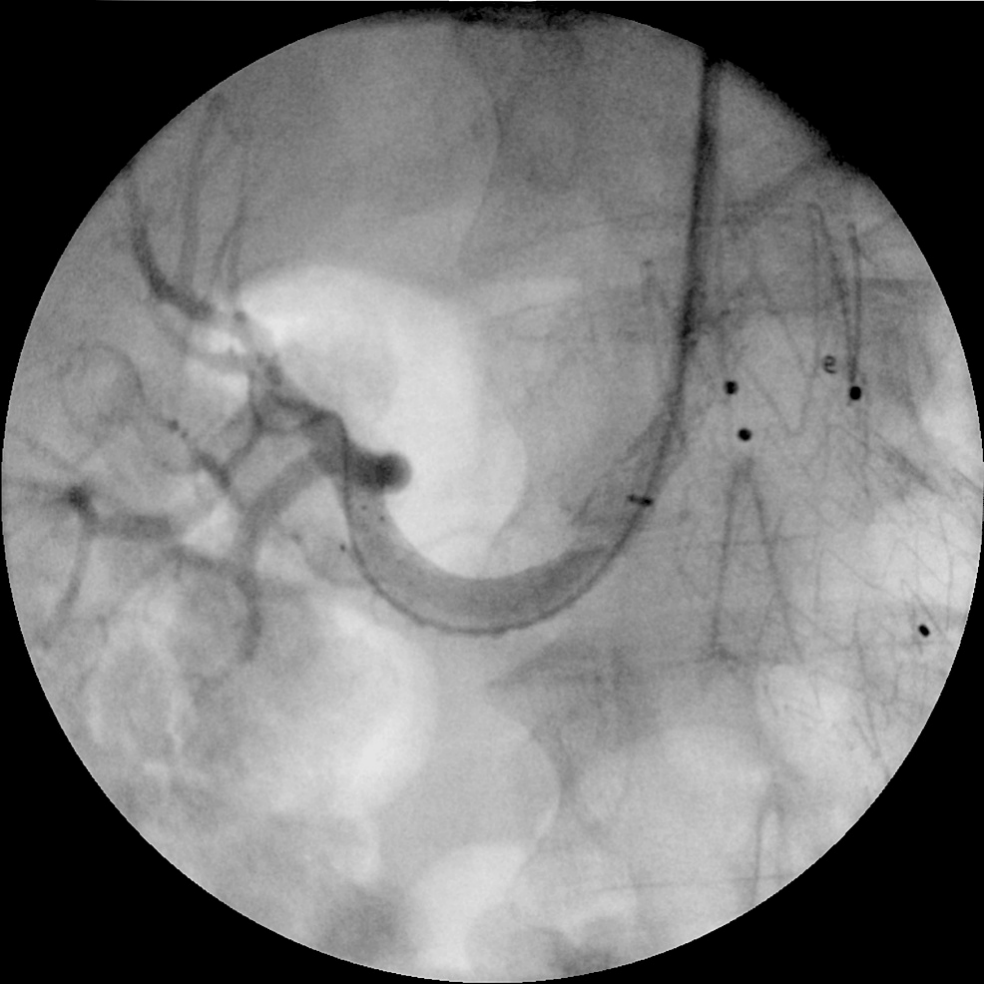
Case 1: Final angiography of recanalization of the right renal stent graft.

The patient was readmitted to our hospital, and he was taken back to the operating theater, where access was obtained through the previous axillary puncture site. A 6 Fr-65 cm sheath was introduced, and with multiple attempts with various catheters, the right renal was eventually cannulated through the occluded Begraft. Angiography verified the renal stent thrombosis. A pre-dilatation angioplasty was conducted using a 5 mm x 100 mm balloon, followed by the deployment of a Protégé 8 mm x 60 mm self-expandable stent (EV3, Plymouth), which overlapped with the thrombosed chimney stent graft. A final angioplasty was carried out using a 7 mm x 60 mm balloon, and the completion angiography revealed satisfactory patency of the right renal artery (Figure 5). The procedure's total duration was 3 hours and 36 minutes, with a fluoroscopy time of 1 hour and 33 minutes, and 300 mL of contrast was administered. The patient recovered well and was discharged on the third postop day with no lumbar pain and normal renal function. The latest follow-up CT scan in one year showed good graft patency and no endoleak.

The second case involved a 72-year-old male with an abdominal juxtarenal aortic aneurysm with a max sack diameter of 52 mm who was also referred to our department from a district hospital. His past medical history was unremarkable involving hypertension and benign hyperplasia of the prostatic gland.

CTA with three-dimensional (3D) reconstruction was performed, and a standard EVAR with a double chimney on both renal arteries was decided due to a short proximal aortic neck.

The patient's position, type of anesthesia, and access were the same as in the previous case. Both renal arteries were cannulated from the axillary artery. The left renal artery was cannulated using a Life Stream (Bard) 6 Fr 40 cm, while the contralateral was also treated using a Life Stream (Bard) 7 Fr 40 cm employing the chimney technique. An Endurant II 32-16-145 mm stent graft (Medtronic Vascular Inc.) main body was inserted from the left femoral artery and deployed with an additional aortic extension (36-36-49 mm), while the contralateral limb was 16-16-93 mm. Final angiography showed good renal and graft patency with no endoleak. The fluoroscopy time for the duration of the procedure was 29 minutes, with a total contrast volume of 220 mL administered. The patient had an uneventful recovery and was discharged on the second postop day with a prescription of clopidogrel 75 mg once daily and normal renal function.

During the 10th postop day, while at home, the patient experienced acute right lumbar pain. A new CTA was performed in a district hospital, revealing bilateral renal thrombosis. The right renal stent graft showed signs of compression or obstruction, while the left renal stent graft did not show signs of kinking or obstruction but displayed slow post-occlusion flow in the distal renal artery. The patient developed acute renal insufficiency with the need for dialysis.

**Figure 6:**
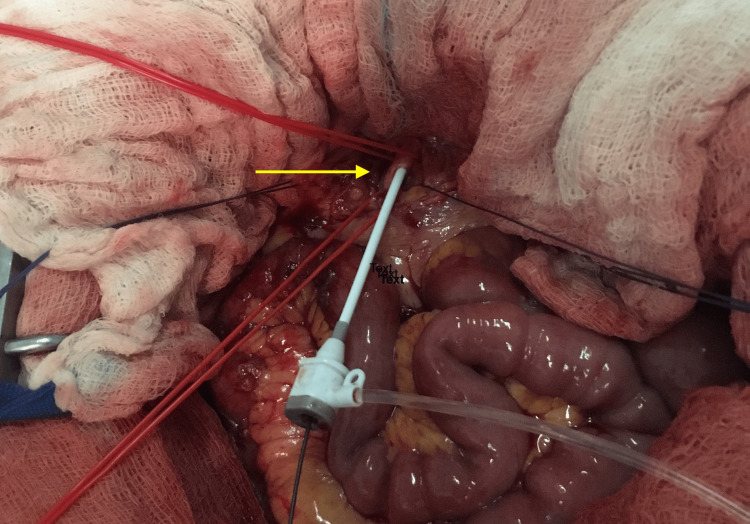
Case 2: Retrograde recanalization of the right renal artery. The arrow shows the puncture site of the right renal artery.

The patient was readmitted to our hospital and taken back to the theater, where access was gained through his previous left axillary puncture site. A 6 Fr 65 cm sheath was introduced, utilizing a 0.035” hydrophilic angled wire. The left renal artery was cannulated, and a self-expandable stent (7 Fr-100 mm) and a balloon-expandable stent (6 Fr-27 mm) were deployed. Angioplasty was conducted using balloons of varying sizes: 5 mm x 100 mm, 5 mm x 60 mm, and 6 mm x 60 mm. Final angiography showed good renal and graft patency. The attempt to recanalize the right renal stent graft was unsuccessful. All materials were removed. Direct pressure hemostasis was applied. On the third postoperative day, the patient was taken back to the operating theater for a second attempt to recanalize the right renal artery. Under local anesthesia, access was gained from the previous left axillary artery with direct puncture. A 6 Fr-65 mm sheath was introduced, and after multiple attempts with various catheters, the intervention was again characterized as unsuccessful. A nephrogram surprisingly showed viable right renal tissue, so a final attempt on the right renal artery was decided. Under general anesthesia, a right subcostal Kocher incision was performed. After mobilizing the gallbladder and the hepatic flexure, access to the retroperitoneal space was gained. The right renal artery was dissected. A 5 Fr-11 cm sheath was introduced, and retrograde cannulation of the right renal artery was attempted with 0.035’’ and 0.014’’ hydrophilic wires (Figure 6). After multiple attempts, the cannulating process was terminated and deemed unsuccessful.

**Figure 7:**
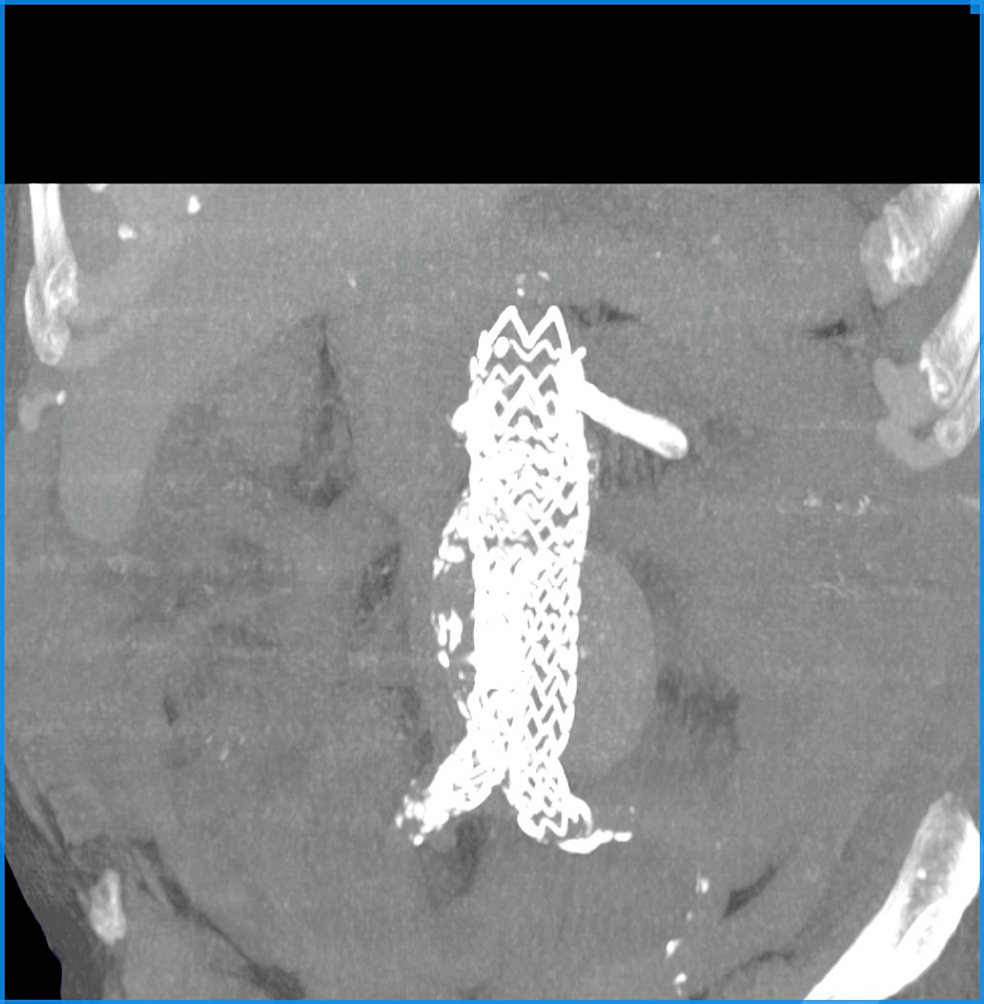
Case 2: CT without contrast showing the stent graft with two chimneys. CT, computed tomography

A postop CT scan without contrast was performed to show the apposition of the stent grafts (Figure 7). The patient had an uneventful recovery and was discharged from the hospital in good overall condition on the 15th postop day, without the need for dialysis. His creatinine level was 1.89 mg/dL, and he was prescribed Salospir 100 mg once daily along with low-molecular-weight heparin on therapeutic dosage for 20 days. The patient is doing well after two years, undergoing renal surveillance with a creatinine level of 2.2 mg/dL. Unfortunately, he is refusing any additional imaging.

## Discussion

Although Medtronic received a CE mark for the Endurant II/IIs stent graft system in late 2016 as an on-label solution for ch-EVAR to treat AAAs with a short proximal neck, there is still significant criticism and a lack of evidence regarding the safety, durability, and extent of complications associated with the technique [4]. Recent guidelines recommend the use of the ch-EVAR as an alternative in the emergency setting or when fenestrated grafts are unavailable [5]. Proximal endoleak (Ia) due to gutter and loss of chimney patency remain the major drawbacks to the wide acceptance of chimney grafts [6]. Furthermore, the technique itself is still evolving, and dedicated equipment is currently under development. The most robust data regarding the chimney technique has been collected from the PERICLES registry, which shows highly promising primary patency of chimney grafts, reaching up to 94% after a mean follow-up of 17 months [7]. Although the initial postoperative outcomes of fenestrated and chimney techniques are similar [8], midterm data comparing fenestrated and branched endografts show statistically significant differences in mortality, endoleaks, renal impairment, and cardiovascular complications in favor of fenestrated grafts [9].

Chimney stent grafts occlude, mostly due to compression from the main body stent graft or kinking at the native orifice of the renal arteries. These defaults can be easily recognized intraoperatively, from follow-up CTA or even plain abdominal X-rays. There are cases, like the one presented, where no specific reason for the occlusion can be identified [10]. A recent study aims to shed light on these cases, asserting that the anatomy in the repair area undergoes constant remodeling in the weeks and months postoperatively [11]. The authors stated that changes in threshold values of cross-sectional area, peak systolic pressure gradient, and peak systolic wall shear stress were significantly associated with chimney graft occlusion and could be added in the postoperative follow-up protocol [11]. Furthermore, the constant displacement and bending of the renal arteries during respiration can affect the structural integrity of any renal stent or stent graft, resulting in thrombosis [12]. Operative factors that seem to improve the results of the technique are a proximal landing zone of 15 mm, aortic stent graft oversizing of 30%, and as few chimneys as possible [13,14].

In the case of acute chimney thrombosis, recanalization should always and urgently be attempted, especially when there is residual slow flow in the distal renal arteries. Nephrons and kidney tissue can still be viable after hours or days of ischemia, and renal function can return to normal values if recanalization is successful [15]. Regarding the recommended approach, open retrograde target vessel catheterization, as described by Oikonomou et al., is feasible. However, it is also associated with higher perioperative morbidity compared to a solely endovascular solution [16]. The latter is always challenging and requires operator experience and patience, long sheath support, multiple different angiographic catheters, wires, and possible long duration of radiation exposure. Thrombectomy of the occluded stent is not mandatory, as in most cases, stent deployment and balloon dilatation ensure excellent angiographic results [17]. Additional stenting should ensure sufficient radial strength and completely cover the length between the aortic flow and the renal artery distally to the chimney graft.

Our experience raises the question of whether all chimney grafts should be supported with a self-expandable stent to avoid possible postop thrombosis. The hypothesis states that using a balloon-expandable covered stent graft supported by a longer bare-metal self-expandable stent could enhance chimney patency rates. Specific stent grafts for ch-EVAR should be designed to facilitate combined material properties of the balloon- and self-expandable stents. In addition, these stent grafts should be available in sufficient lengths [18]. Another issue that has not been addressed in the chimney technique is the ideal orientation of the stent graft as it protrudes in the aorta and how this can be technically achieved. Finally, there is no robust data regarding the role of antiplatelets and anticoagulation in patients undergoing ch-EVAR.

## Conclusions

The ability to utilize the chimney technique under local anesthesia in a wide variety of aortic anatomies, coupled with the fact that most procedures can be performed using inventory already in place in most endovascular theaters, makes this therapeutic approach still quite attractive. This is more obvious in emergency cases where open repair has higher rates of morbidity and mortality and there is no time for custom-made fenestrated or branched devices. The low early mortality and complication rates seem advantageous and create the foundation to identify the ideal anatomies and device combinations for better long-term results. In the case of stent graft thrombosis, early diagnosis, and treatment, although laborious, can prevent permanent renal impairment.
